# Impact of COVID-19 after lung transplantation: A retrospective multicenter comparison of clinical outcomes in Denmark and Sweden

**DOI:** 10.1016/j.jhlto.2025.100377

**Published:** 2025-08-20

**Authors:** Embla Bodén, Michael Perch, Regitze Hertz Liebermann, John Mackay Søfteland, Jesper M. Magnusson, Sandra Lindstedt

**Affiliations:** a Department of Clinical Sciences, Lund University, Lund, Sweden; b Wallenberg Center for Molecular Medicine, Lund University, Lund, Sweden; c Lund Stem Cell Center, Lund University, Lund, Sweden; d Department of Cardiology, Section for Lung Transplantation, Rigshospitalet, Copenhagen, Denmark; e Department of Clinical Medicine, University of Copenhagen, Copenhagen, Denmark; f The Transplant Institute, Sahlgrenska University Hospital, Gothenburg, Sweden; g Department of Surgery, Institute of Clinical Sciences, Sahlgrenska Academy, University of Gothenburg, Gothenburg, Sweden; h Department of Pulmonology, Institute of Medicine, Sahlgrenska Academy, University of Gothenburg, Gothenburg, Sweden; i Department of Cardiothoracic Surgery and Transplantation, Skåne University Hospital, Lund, Sweden

**Keywords:** lung transplantation, COVID-19, sociopolitical approach, outcome, Denmark, Sweden

## Abstract

**Background:**

The coronavirus disease-2019 (COVID-19) pandemic posed pronounced challenges in the care of lung transplant (LTx) recipients. Global variations in containment strategies and the introduction of messenger ribonucleic acid (mRNA) vaccines have sparked extensive debate in both scientific and public arenas.

**Methods:**

This retrospective study compared outcomes among LTx recipients in Denmark, which implemented a more restrictive COVID-19 containment strategy, and Sweden, which adopted a less restrictive approach. A total of 318 LTx recipients with at least 1 episode of polymerase chain reaction (PCR)-confirmed COVID-19 were included. Propensity score weighting was applied to balance covariates, and survival outcomes were analyzed using weighted Cox proportional hazards and Kaplan-Meier analyses.

**Results:**

No significant differences in mortality or risk of chronic lung allograft dysfunction (CLAD) were found between countries (hazard ratios [HR] for death, Sweden = 1.49, 95% confidence intervals [CI]: 0.68-3.26, *p* = 0.314; HR for CLAD, Sweden = 0.63, 95% CI: 0.32-1.25, *p* = 0.187). Unvaccinated patients had a significantly higher risk of death compared to vaccinated patients (HR = 3.49, 95% CI: 1.46-8.34, *p* = 0.005), and infections with the original Wuhan strain carried a higher risk than Omicron (HR = 3.59, 95% CI: 1.53-8.44, *p* = 0.003). CLAD development or progression was not significantly associated with any subgroup.

**Conclusions:**

Despite differences in timing of infections and case load between Sweden and Denmark, clinical outcomes among infected LTx recipients were comparable. mRNA vaccination was strongly associated with improved survival. The results of the current study highlight the importance of continued vaccination efforts and tailored containment strategies in vulnerable populations.

## Background

A lung transplant (LTx) is currently the only definitive treatment option for patients with end-stage pulmonary disease. Meanwhile, the long-term survival rates following LTx are the lowest of any solid organ transplantation, with 5-year survival rates between 50% and 60%.^1^, ^2^ Chronic lung allograft dysfunction (CLAD), followed by infectious complications, represents the primary limiting factor for long-term survival in these patients.^3^

The emergence of the severe acute respiratory syndrome coronavirus 2 (SARS-CoV-2), an enveloped, positive-sense single-stranded ribonucleic acid virus, which causes coronavirus disease-2019 (COVID-19), has complicated the clinical management of lung transplant recipients, particularly due to their heightened immunosuppression and infection risk.^4^, ^5^, ^6^ COVID-19 ranges in severity from asymptomatic infection to severe respiratory failure, including acute respiratory distress syndrome *(*ARDS), multiorgan failure, and death. Common clinical symptoms include dyspnea, cough, pneumonia, fever, fatigue, myalgia, and gastrointestinal disturbances.^7^, ^8^ Declared a global pandemic by the World Health Organization in March 2020, COVID-19 rapidly became a global public health crisis.^9^, ^10^, ^11^ The virus spreads via respiratory droplets from coughing or sneezing and indirectly via contaminated surfaces.^5^ SARS-CoV-2 infection severity correlates with cytokine storm intensity, significantly influencing disease outcomes.^6^, ^12^ Viral mutations led to multiple SARS-CoV-2 variants with distinct clinical profiles.^13^ The earlier Wuhan, Alpha, and Delta variants were associated with similar severity levels, inducing high proinflammatory responses.^14^ Conversely, the later Omicron variant caused relatively milder disease due to less pronounced cytokine activation.^14^

The global spread of COVID-19 had far-reaching implications for all regions, including Scandinavia. The neighboring countries Sweden and Denmark share cultural similarities, comparable societal structures, and analogous health care systems.^15^ Despite these commonalities, their national strategies to limit COVID-19 transmission differed substantially. Denmark, like many European countries, adopted mandatory restrictions, including lockdowns, enforced social distancing, compulsory face masks, and widespread testing. In contrast, Sweden employed primarily voluntary guidelines for the general population.^15^, ^16^, ^17^ Both approaches received significant international attention; Sweden's less restrictive strategy faced criticism during the pandemic’s peak, while Denmark’s strict measures have since sparked debate regarding their long-term societal consequences.^18^

The rapid development and distribution of vaccines significantly mitigated COVID-19's global impact.^19^ By 2023, nearly 5 billion people worldwide had been fully vaccinated. The most widely utilized vaccines were the messenger ribonucleic acid (mRNA)-based vaccines BNT162b2 (Pfizer-BioNTech) and mRNA-1273 (Moderna), which received emergency use authorization from the Food and Drug Administration in December 2020.^8^ These vaccines demonstrated robust efficacy (70%-95%) in the general population, effectively stimulating both humoral and cellular immune responses, distinct from traditional protein-based vaccines, which primarily induce antibody responses.^8^, ^20^, ^21^, ^22^

This study aims to identify factors influencing COVID-19 outcomes among Scandinavian LTx recipients. Given the marked differences in COVID-19 management strategies between Sweden and Denmark, we specifically sought to evaluate whether these national approaches impacted outcomes in lung transplant recipients.

## Methods

### Study design

This retrospective multicenter study included lung transplant (LTx) recipients from 2 Swedish centers (Lund and Gothenburg) and 1 Danish center (Copenhagen) who had at least 1 PCR-confirmed episode of COVID-19 post-transplantation. Patients who underwent single lung, double lung, heart-LTx, or lung retransplantation between 1993 and 2023 and who were alive as of February 1, 2020, were included. Follow-up extended for 1 year (365 days) after the initial positive polymerase chain reaction (PCR)-test for SARS-CoV-2 infection or until death, whichever occurred first. COVID-19–related deaths were defined as cases where COVID-19 was listed as the primary or contributing cause of death. Definitions were applied uniformly across cohorts. All included patients were aged 18 years or older.

**Figure 1:**
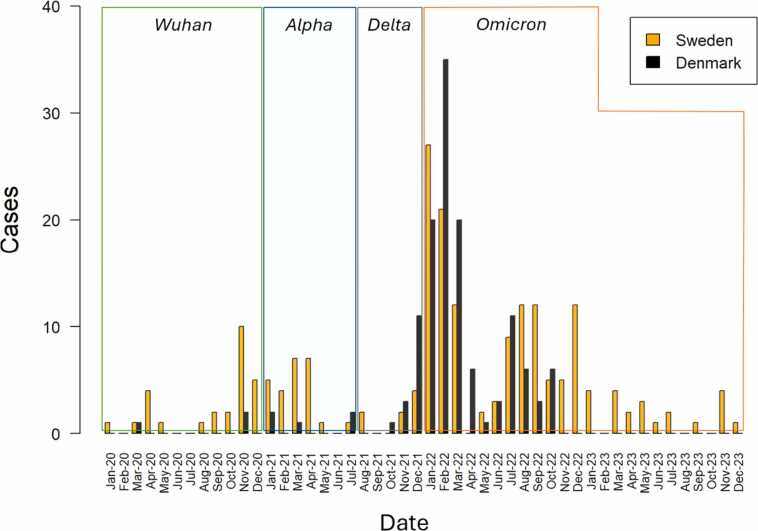
Incidence of the different strains of SARS-CoV-2 over time. Bar plot showing the number of cases of COVID-19 per country over time (orange = Sweden, black = Denmark) as well as the dominating strain of SARS-CoV-2 in the 4 different time periods (Wuhan: January 2020-December 2020, Alpha: January 2021-July 2021, Delta: August 2021-December 2021, Omicron: January 2022-December 2023). COVID-19, coronavirus disease-2019; SARS-CoV-2, severe acute respiratory syndrome coronavirus 2.

Cases were categorized according to the dominant circulating SARS-CoV-2 variant at the time of infection, following the timeline established by Yewdell.^23^ The variants and corresponding dominant periods were defined as Wuhan (January-December 2020), Alpha (January-July 2021), Delta (August-December 2021), and Omicron (January 2022-December 2023) (Figure 1).

Full vaccination against SARS-CoV-2 was defined as receiving at least 2 doses of any vaccine against COVID-19.

CLAD was defined according to the International Society for Heart and Lung Transplantation’s consensus, as a persistent decline in forced expiratory volume in 1 second (FEV1) of 20% or more, compared to baseline values where all other causes were excluded.^7^ CLAD was recorded if present before COVID-19, and development of or progression to a higher CLAD grade was recorded within the follow-up time. Surveillance bronchoscopy practices during the pandemic varied between centers and were not consistently documented. As such, their potential influence on CLAD detection or management could not be assessed.

Indications for LTx were categorized as either obstructive (including chronic obstructive pulmonary disease, alpha-1-antitrypsin deficiency, and emphysema), restrictive (including all types of pulmonary fibrosis, rheumatoid arthritis, systemic sclerosis, and hemosiderosis), vascular (including pulmonary arterial hypertension and congenital heart defects), cystic fibrosis, or other (including lymphangioleiomyomatosis, graft vs host disease, primary ciliary dysfunction, ARDS, pneumonitis, and antisynthetase syndrome).

Annual estimates of the total number of lung transplant recipients alive during the study period (2020-2023) were obtained from Scandiatransplant.

The study was conducted in accordance with the Declaration of Helsinki and approved by the Swedish Ethical Review Authority (Dnr: 2020-02153, 2020-01771).

### Data sourcing

Data on demographics, clinical course, vaccination status, therapeutic interventions, and clinical outcomes after COVID-19 infection were collected through retrospective review of electronic medical records (Table 1).

**Table 1:** Demographics Divided by Center of Inclusion

	Lund	Gothenburg	Copenhagen	All
N (% of all)	56 (17)	132 (42)	130 (41)	318 (100)
Age at diagnosis (years), mean (range)	54 (23-75)	55 (18-78)	56 (20-74)	55 (18-78)
Male sex, *n* (%)	28 (50)	60 (45)	67 (52)	155 (49)
Height (cm), mean (range)	169 (148-191)	169 (145-194)	171 (143-190)	170 (143-194)
Weight (kg), mean (range)	69 (31-119)	74 (34-127)	76 (40-134)	74 (31-134)
BMI (kg/m^2^), mean (range)	24.1 (14.2-42.3)	25.8 (13.8-42.4)	25.9 (15.1-40.2)	25.5 (13.8-42.4)
CCI score				
0, *n* (%)	3 (5)	19 (14.5)	13 (10)	35 (11)
1, *n* (%)	5 (9)	14 (10.5)	22 (17)	41 (13)
2, *n* (%)	7 (13)	21 (16)	37 (28)	65 (20.5)
3, *n* (%)	10 (18)	21 (16)	27 (21)	58 (18)
4, *n* (%)	10 (18)	15 (11.5)	16 (12)	41 (13)
5, *n* (%)	8 (14)	18 (13.5)	13 (10)	39 (12.5)
6, *n* (%)	4 (7)	8 (6)	1 (1)	13 (4)
7, *n* (%)	4 (7)	8 (6)	1 (1)	13 (4)
8, *n* (%)	0 (0)	5 (4)	0 (0)	5 (1.5)
9, *n* (%)	4 (7)	3 (2)	0 (0)	7 (2)
10, *n* (%)	1 (2)	0 (0)	0 (0)	1 (0.5)
Type of transplantation				
Single, *n* (%)	3 (5)	12 (9)	16 (13)	31 (10)
Double, *n* (%)	42 (75)	105 (80)	106 (81)	253 (79)
Heart and lung, *n* (%)	2 (4)	3 (2)	5 (4)	10 (3)
Retransplantation, *n* (%)	9 (16)	12 (9)	3 (2)	24 (8)
CMV status			a	
D+/R−, *n* (%)	8 (14)	15 (11)	19 (15)	42 (13)
D+/R+ or D−/R+, *n* (%)	46 (82)	105 (80)	48 (37)	199 (63)
D−/R−, *n* (%)	2 (4)	12 (9)	15 (12)	29 (9)
Underlying condition				
CF, *n* (%)	13 (23)	16 (12)	15 (12)	44 (14)
Obstructive pulmonary disease, *n* (%)	19 (34)	33 (25)	51 (39)	103 (32)
Restrictive pulmonary disease, *n* (%)	17 (30)	65 (49)	51 (39)	131 (41)
Pulmonary vascular disease, *n* (%)	6 (11)	8 (6)	9 (7)	23 (7)
Other, *n* (%)^b^	1 (2)	10 (8)	4 (3)	15 (5)
Infecting strain of SARS-CoV-2				
Wuhan, *n* (%)	6 (11)	20 (15)	3 (2)	29 (9)
Alpha, *n* (%)	8 (14)	18 (14)	5 (4)	31 (10)
Delta, *n* (%)	4 (7)	5 (4)	15 (12)	24 (7.5)
Omicron, *n* (%)	38 (68)	89 (67)	107 (82)	234 (73.5)
Number of COVID-19 infections				
1, *n* (%)	51 (91)	122 (92)	126 (97)	299 (94)
2, *n* (%)	5 (9)	9 (7)	3 (2)	17 (5)
3, *n* (%)	0 (0)	1 (1)	1 (1)	2 (1)
Incomplete vaccination before COVID-19, *n* (%)	10 (18)	33 (25)	6 (5)	49 (15)
Days from LTx to COVID-19, mean (range)	2,492 (1-9,974)	2,352 (28-9,027)	2,881 (72-10,613)	2,607 (1-10,613)
Days from COVID-19 to end of follow-up, mean (range)	323 (2-365)	340 (6-365)	344 (18-365)	338 (2-365)
CLAD stage before infection				
0, *n* (%)	38 (68)	117 (88)	105 (81)	260 (82)
1, *n* (%)	9 (16)	5 (4)	5 (4)	19 (6)
2, *n* (%)	6 (11)	5 (4)	8 (6)	19 (6)
3, *n* (%)	3 (5)	5 (4)	12 (9)	20 (6)
CLAD stage at end of follow-up				
0, *n*^c^	36 (64)	105 (80)	89 (68)	230 (72)
1, *n*	9 (16)	9 (6.5)	10 (8)	28 (9)
2, *n*	6 (11)	8 (6)	13 (10)	27 (8.5)
3, *n*	5 (9)	10 (7.5)	18 (14)	33 (10.5)
FEV1 (liter) before infection, mean (range)	1.78 (0.6-2.79)	2.20 (0.53-4.49)	2.24 (0.40-5.77)	2.14 (0.40-5.77)
FEV1 (liter) at end of follow-up, mean (range)	1.8 (0.65-3.90)	2.15 (0.74-4.19)	2.14 (0.51-4.34)	2.09 (0.51-4.34)

Abbreviations: ARDS, acute respiratory distress syndrome; BMI, body mass index, CCI, Charlson comorbidity index; CF, cystic fibrosis; cm, centimeters; CMV, cytomegalovirus; COVID-19, coronavirus disease-2019; D, donor; GvHD, graft vs host disease; kg, kilos; LAM, lymphangioleiomyomatosis; LTx, lung transplantation; N, number of; R, recipient; SARS-CoV-2, severe acute respiratory syndrome coronavirus 2.

Data presented as number of subjects, percentage, mean, and range.

a CMV status was not available for 48 of the subjects from Copenhagen.

b Other underlying pulmonary conditions include LAM, histiocytosis X, antisynthetase syndrome, primary ciliary dyskinesia, GvHD, ARDS, hypersensitivity pneumonitis, and primary ciliary dysfunction.

c Patients without a CLAD diagnosis at end of follow-up or death.

### Statistical analysis

All statistical analyses were carried out in R version 4.1.2 (November 1, 2021). Demographic data are presented as number of (*n*), percentage, mean, and range. Subgroups (Sweden vs Denmark, vaccinated vs unvaccinated, early vs late COVID-19 infection) were balanced via propensity score weighting based on sex, Charlson Comorbidity Index at the time of infection (International Classification of Diseases (ICD)-10–based algorithm by Quan et al), preinfection CLAD status, and transplantation center.^8^, ^9^ Weighted Kaplan-Meier curves were fitted to visualize survival and CLAD occurrence, with the events being death/graft failure or development/progression of CLAD. The distribution of dominant SARS-CoV-2 strains by country was visualized in a bar plot (Supplemental Figure 4). Study subjects that were alive or without changes in CLAD status at the end of follow-up were censored. Weighted Cox proportional hazards models were applied to analyze differences in survival and CLAD-free survival between the subgroups, yielding hazard ratios (HR) with 95% confidence intervals (CI). CLAD-free survival is an established and widely applied end-point in LTx outcome studies.^10^, ^11^ To visualize the timing of COVID-19 infection across countries, a cumulative incidence plot stratified by country was generated and is provided in the Supplemental Material (Supplemental Figure 3). Differences in delta FEV1 and FEV1 variability between countries were assessed with *t*-test and Levene’s test. Statistical significance was defined as *p* < 0.05.

## Results

### Descriptive results

A total of 318 LTx recipients from Lund, Sweden (*n* = 56), Gothenburg, Sweden (*n* = 132), and Copenhagen, Denmark (*n* = 130) were included. The mean age was 55 years (range 18-78 years). The cohort included 155 males (49%), with restrictive and obstructive pulmonary diseases being the most common indications for transplantation. Most of the included patients underwent double LTx (*n* = 253, 79%). The mean time elapsed from LTx to COVID-19 was 7.1 years (2,607 days, range 1-10,613) and the mean time from LTx to end of follow-up or death was 7.9 years (2,889 days, range 283-10,978). The mean time from COVID-19 to end of follow-up or death was 338 days (range 2-365). A total of 33 patients died within the follow-up time (Sweden, *n* = 23; Denmark, *n* = 10), corresponding to 10% of the total study population, and 12.4% and 7.6% in each country’s population of infected LTx recipients, respectively. Thirty-six patients developed or progressed in CLAD during the follow-up period, making up 11% of the cohort. Among patients with CLAD stage 0 at baseline (*n* = 260), 30 (11.54%) developed CLAD during follow-up. Among patients with existing CLAD before infection (stages 1-3, *n* = 58), 6 (10.34%) progressed to a higher grade. The total number of LTx recipients who were alive and at risk of infection by SARS-CoV-2 during the pandemic in Sweden and Denmark remained relatively consistent over the years (2020: Sweden, *n* = 540, Denmark, *n* = 276; 2021: Sweden, *n* = 537, Denmark, *n* = 275; 2022: Sweden, *n* = 533, Denmark, *n* = 267; 2023: Sweden, *n* = 528, Denmark, *n* = 272). Using these national cohort estimates from 2020-2023, the average annual infection rate among LTx recipients was 8.8% in Sweden and 11.9% in Denmark. The corresponding population-wide mortality rates were 4.3% and 3.7%, respectively.

### No significant difference in death or CLAD development between Sweden and Denmark

**Figure 2:**
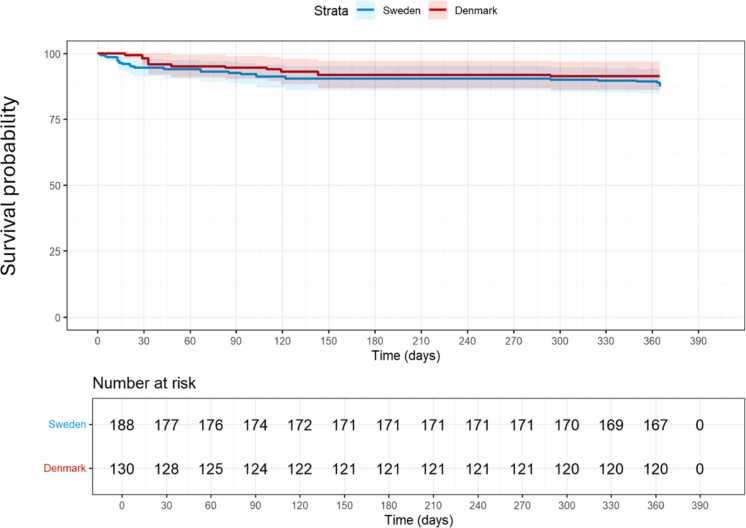
No difference in the risk of death between Sweden and Denmark. Kaplan-Meier curve displaying survival probability with 95% CI for patients with COVID-19 in Sweden and in Denmark (blue = Sweden, red = Denmark). There is no statistically significant difference in survival compared between Swedish and Danish patients (*p* = 0.314). A table with numbers at risk for every 30 days is placed below the curve. CI, confidence interval; COVID-19, coronavirus disease-2019.

The patients were divided into subgroups depending on country of residence (Sweden, *n* = 188 and Denmark, *n* = 130). Further subdivisions were made for additional analyses, including vaccination status at date of positive PCR-test (vaccinated, *n* = 282 and unvaccinated, *n* = 36) and period of infection (early [January 2020-December 2021], *n* = 83 and late (January 2022-December 2023), *n* = 235). Survival from the date of positive PCR-test did not differ significantly between Sweden and Denmark (HR for death, Sweden vs Denmark = 1.49, 95% CI: 0.68-3.26, *p* = 0.314; Figure 2). Similarly, no significant difference in CLAD development or progression risk was observed between the countries (HR for Sweden vs Denmark = 0.63, 95% CI: 0.32-1.25, *p* = 0.187; Supplemental Figure 1).

### Increased risk of death for patients infected with the Wuhan strain of SARS-CoV-2

Patients were divided into 4 subgroups depending on the dominant strain of SARS-CoV-2 at time of infection. Patients infected during the Wuhan strain period had a significantly higher risk of death compared to those infected during the Omicron period (HR = 3.59, 95% CI: 1.53-8.44, *p* = 0.003). We also compared the incidence of CLAD development and progression between patients affected by the 4 different strains of SARS-CoV-2 but found no significant differences.

### Lower survival rates after COVID-19 for unvaccinated patients

**Figure 3:**
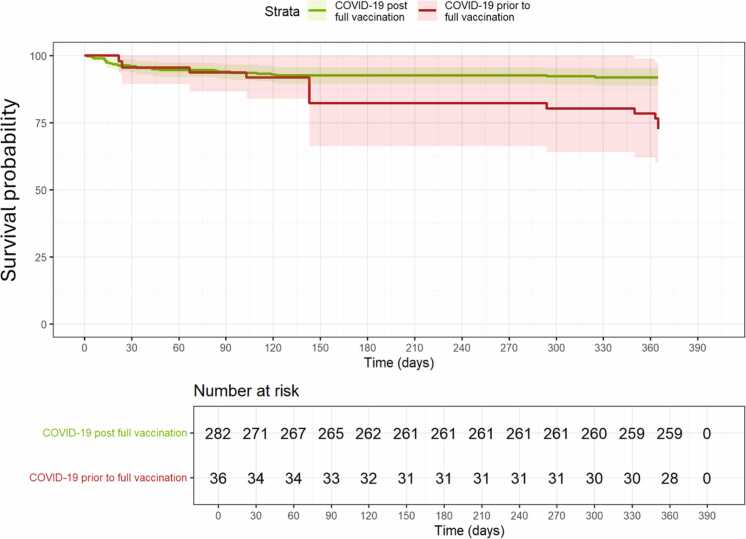
Significantly higher risk of death for unvaccinated patients. Kaplan-Meier curve displaying survival probability with 95% CI for patients with COVID-19 before vs after full vaccination (red = unvaccinated, green = vaccinated). There is a statistically significant increase in the risk of death for unvaccinated patients compared to vaccinated patients (*p* = 0.005). A table with numbers at risk for every 30 days is placed below the curve. CI, confidence interval; COVID-19, coronavirus disease-2019.

The mRNA vaccines against SARS-CoV-2 were introduced in both Sweden and Denmark on December 27, 2020. To evaluate the efficiency of vaccination, survival rates from the date of positive PCR-test to the end of follow-up were compared between patients who tested positive before (*n* = 36) vs after (*n* = 282) full vaccination. Unvaccinated patients had significantly higher mortality compared to vaccinated patients (HR = 3.49, 95% CI: 1.46-8.34, *p* = 0.005; Figure 3). Stratifying by country, this increased risk remained significant for unvaccinated Swedish patients compared to vaccinated patients from both Sweden (HR = 3.55, 95% CI: 1.54-8.22, *p* = 0.003) and Denmark (HR = 4.56, 95% CI: 1.81-11.46, *p* = 0.001).

### No increased risk of CLAD development or progression for unvaccinated patients

CLAD development or progression occurred in 36 patients within the year following COVID-19. There was no significant difference in the risk of CLAD development or progression between unvaccinated and vaccinated patients (HR = 1.87, 95% CI: 0.57-6.11, *p* = 0.303; Supplemental Figure 2). A comparison of the survival between patients with and without development or progression of CLAD was also done, revealing no statistically significant increase in short-term mortality among patients who developed or progressed in CLAD after COVID-19 (HR = 1.20 (95% CI: 0.41-3.47), *p* = 0.740).

To further assess functional changes after infection with COVID-19, we analyzed individual-level changes in FEV1 from baseline to end of follow-up. While the mean change in FEV1 did not differ significantly between countries (mean ΔFEV1, liter: Denmark = −0.142, Sweden = 0.001; *p* = 0.117), Levene’s test revealed a statistically significant difference in variance between groups (*p* < 0.001). A raincloud plot illustrating this distribution is provided in Supplemental Figure 5.

## Discussion

LTx remains the final treatment option for end-stage pulmonary disease, but the procedure demands much of the patient.^12^ Comparatively high doses of immunosuppressive drugs render LTx recipients highly susceptible to infections, and the management of this patient group posted significant challenges during the COVID-19 pandemic.^13^, ^14^ Sociopolitical strategies varied significantly across Europe, with Denmark adopting stricter mandatory restrictions, while Sweden favored voluntary recommendations and maintained a relatively open society. These contrasting strategies led to ongoing scrutiny of Sweden’s less restrictive approach, particularly regarding its long-term health and societal outcomes.^28^, ^29^ Denmark’s restrictions limited in-person work, schooling, nonhousehold social interactions, and access to medical appointments.^15^ In Sweden, schools and public spaces were kept mostly open and the public was instructed on the importance of self-imposed restrictions. Previous research shows that the extent to which social contacts were limited was similar between both countries, and it has been theorized that recommendations may be equally as effective as mandatory restrictions.^15^, ^16^ Additionally, the sense of loneliness has been revealed to be elevated in countries with high levels of restrictiveness during the pandemic.^17^, ^18^ The observed infection rates align with previous national data on COVID-19 incidence in LTx recipients.^19^

Multiple factors influence reported mortality rates, including effectiveness of public health measures, accuracy of case identification and death registration, and accessibility and quality of medical care. Previously published research has shown that the in-hospital mortality rates for patients admitted to the intensive care unit due to COVID-19 vary between 15% and 40% in different parts of the world, and that the death rates for LTx recipients in specific lie between 8% and 55%.^19^, ^20^, ^21^, ^22^ In the current study, the mortality rate was 10% for all included patients, which is among the lowest numbers reported. Furthermore, the current data reveal no significant differences in mortality or CLAD development between Swedish and Danish patients (Figure 2, Supplemental Figure 1). While population-wide mortality for the full study period (2020-2023) was quite similar (4.3% in Sweden and 3.7% in Denmark), we also present data from 2020-2021 to allow direct comparison with prior studies and to allow comparison during the period when national containment strategies differed the most. During the early phase 2020-2021, the population-wide mortality rate in Sweden was higher (1.7%) than Denmark’s (0.7%). These estimates were derived using population data from Scandiatransplant, which tracks transplant activity across both countries. A previous national Swedish study reported an even higher mortality rate of 2.2% during the same period.^33^ This discrepancy may reflect differences in data sources and case definitions: the national study included both PCR-confirmed and ICD-coded COVID-19 cases but did not include cases transplanted during the pandemic. In contrast, our cohort was limited to PCR-confirmed infections but included newly transplanted cases. Although the size of the LTx population in both countries remained stable over time, variations in testing intensity and surveillance practices may have further influenced reported rates.

During the pandemic, SARS-CoV-2 mutated several times, giving rise to different distinct strains, a phenomena called antigenic drift.^23^ When comparing survival and CLAD-free survival between patients infected with the different strains of SARS-CoV-2, we found a significantly increased risk of death for patients infected during the period when the earliest strain *Wuhan* was dominant compared to patients infected by the latest strain *Omicron*. This is corroborated by previous research, stating that later waves of COVID-19 resulted in lower mortality rates compared to earlier ones.^24^ It has furthermore been shown that the *Omicron* variant of SARS-CoV-2 causes less severe infections but has an inherently higher transmissibility compared to other strains of the virus.^25^ Strain distribution differed slightly between countries, with a higher proportion of infections during the Wuhan and Alpha periods in Sweden, and more Omicron infections in Denmark. These patterns likely reflect national differences in epidemic timing and transmission dynamics (Supplemental Figure 4). Beyond antigenic drift, declining mortality over time was likely influenced by increased vaccine coverage and growing clinical experience.^24^ No significant differences in CLAD-free survival were identified between patients infected by different SARS-CoV-2 variants (data not shown but available on request).

The quick introduction of mRNA vaccines played a vital role in the battle against COVID-19. LTx recipients show markedly lower humoral response rates to this type of vaccination compared to the general population, with 2 doses yielding response rates below 40%.^13^, ^14^ The lowered response rates are thought to be caused by the simultaneous treatment with antimetabolites, which is common in these patients.^26^ Moreover, the antibody titers following vaccination of LTx recipients decline faster over time. This limited response has been partly offset by booster doses.^13^ We assessed the effect of ≥2 vaccine doses on mortality and CLAD risk. The results revealed a statistically significant reduction in the risk of death for vaccinated patients compared to unvaccinated patients (Figure 3). This effect persisted in country-stratified analyses, with unvaccinated Swedish patients experiencing higher mortality than both Swedish and Danish vaccinated counterparts. We found no significant difference in CLAD development or progression between vaccinated and unvaccinated groups (Supplement Figure 2); however, due to the competing risk of death, this result requires cautious interpretation. Our results indicate a significant positive effect of at least 2 doses of mRNA vaccine against severe COVID-19, which is in line with a previously published article by Moghadas et al, showing that both the Pfizer and the Moderna vaccines reduce hospitalization and death rates among patients affected by COVID-19 in the United States.^27^

Severe respiratory tract infections can reduce lung function in LTx recipients, often measured as a decline in FEV1 or forced vital capacity.^28^, ^29^ A persistent decrease in lung function can result in a CLAD diagnosis, a complication previously linked to COVID-19.^30^ In the current study, 11% of the cohort developed or progressed in CLAD within the first year after COVID-19. These numbers are slightly lower compared to the estimated 1-year CLAD incidence in other parts of Scandinavia of 14%.^31^ However, we did not assess postinfection FEV1 changes relative to individual pre-COVID baselines, which may have limited the detection of functional impairments not meeting formal CLAD criteria. Furthermore, it is important to acknowledge that mortality may act as a competing risk for CLAD development, particularly in patients who died shortly after infection. While our primary analysis used Cox proportional hazards models for consistency across outcomes, this approach does not account for the possibility that patients may have died before CLAD could develop or be detected. This may have led to an underestimation of CLAD incidence, especially in higher-mortality subgroups. The lower incidence of CLAD may reflect a predominance of long-term recipients and reduced circulation of other respiratory viruses during the pandemic.^32^ Although the mean decline in FEV1 after COVID-19 was similar between countries, we observed significantly greater variability in Denmark compared to Sweden. This suggests that while average outcomes were comparable, the clinical trajectory following infection may have been more heterogeneous in Danish patients, potentially reflecting differences in comorbidity burden, post-COVID care, or local rehabilitation practices. The high CLAD-free survival rates in the current study support the findings that COVID-19 in LTx recipients does not negatively affect graft function within the first year after infection, which has also been shown by others.^29^, ^33^

While lockdowns and other nonpharmaceutical interventions effectively curbed viral spread, recent studies have drawn attention to their unintended societal and health-related consequences. These include substantial negative impacts on mental health, physical activity, and obesity, disproportionately affecting vulnerable groups and intensifying existing health inequalities.^34^ Additionally, increased anxiety, depression, stress disorders, and reduced access to health care and social services were broadly reported, underscoring the complexity and trade-offs inherent in pandemic response measures.^35^

In the current study, we found no differences in mortality or CLAD development between Swedish and Danish LTx recipients despite differing sociopolitical tactics during the COVID-19 pandemic. However, patients in both countries have likely practiced varying degrees of self-isolation regardless of legal reinforcements. While our study did not assess mental health, physical activity, or social equity directly, previous research has reported that restrictive public health measures can negatively impact these aspects, which should be considered when evaluating the broader consequences of pandemic policies. These findings offer valuable clinical insight but must be interpreted with caution, particularly due to the small number of unvaccinated cases and potential differences in national testing and reporting practices. An important limitation of this study is the relatively small number of events, especially in subgroup analyses. This may have limited the power to detect smaller differences in mortality and CLAD incidence between countries. The absence of statistically significant findings should therefore be interpreted with caution. Additionally, although annual numbers regarding the total LTx population in each country were available, individual-level data for the full cohort were not acquired. As a result, it was not possible to calculate cumulative infection and mortality rates relative to the total at-risk population over time. We also did not compare mortality rates with rates of the prepandemic era. Data on acute cellular rejection were not systematically collected in this study, and their potential impact on CLAD development has therefore not been assessed. Similarly, data on specific COVID-19 treatments were not collected, as treatment protocols evolved throughout the pandemic and varied across centers, and such data were unlikely to significantly affect the interpretation of outcomes. Larger, long-term studies with matched cohorts are needed to confirm these trends.

## Conclusions

Among lung transplant recipients with PCR-confirmed COVID-19, clinical outcomes—including mortality and CLAD development—were comparable between Sweden and Denmark, despite differences in the timing of infections and overall national case burden. Vaccination was strongly associated with improved survival in this high-risk population. These findings highlight the importance of continued vaccination efforts and tailored protection strategies for immunosuppressed individuals.

## Author contributions

Conceptualization: S.L., M.P., and J.M.; methodology: S.L., M.P., and J.M.; project administration: S.L. and J.M.; data curation: E.B. and R.H.L.; formal analysis: E.B.; software: E.B.; investigation: E.B. and R.H.L.; validation: E.B.; writing – original draft: E.B., M.P., R.H.L., J.M.S., J.M., and S.L.; writing – review and editing: E.B., M.P., R.H.L., J.M.S., J.M., and S.L.; resources: S.L., M.P., J.M.S., and J.M.; visualization: E.B.; supervision, S.L. and J.M.; funding acquisition: S.L., M.P., J.M.S., and J.M. All authors have read and agreed to the published version of the manuscript.

## Disclosure statement

The authors declare that they have no known competing financial interests or personal relationships that could have appeared to influence the work reported in this paper.

## Footnotes

Appendix A Supplementary data associated with this article can be found in the online version at doi:10.1016/j.jhlto.2025.100377.
