# Supplementary material for: Impact of COVID-19 after lung transplantation: A retrospective multicenter comparison of clinical outcomes in Denmark and Sweden
Source: JHLT Open. 2025 Aug 20;10:100377. doi: 10.1016/j.jhlto.2025.100377 (PMC12446544; doi:10.1016/j.jhlto.2025.100377)
Supplement: Supplementary file 1 — Supplementary material [file mmc1.docx]

**Supplemental material**


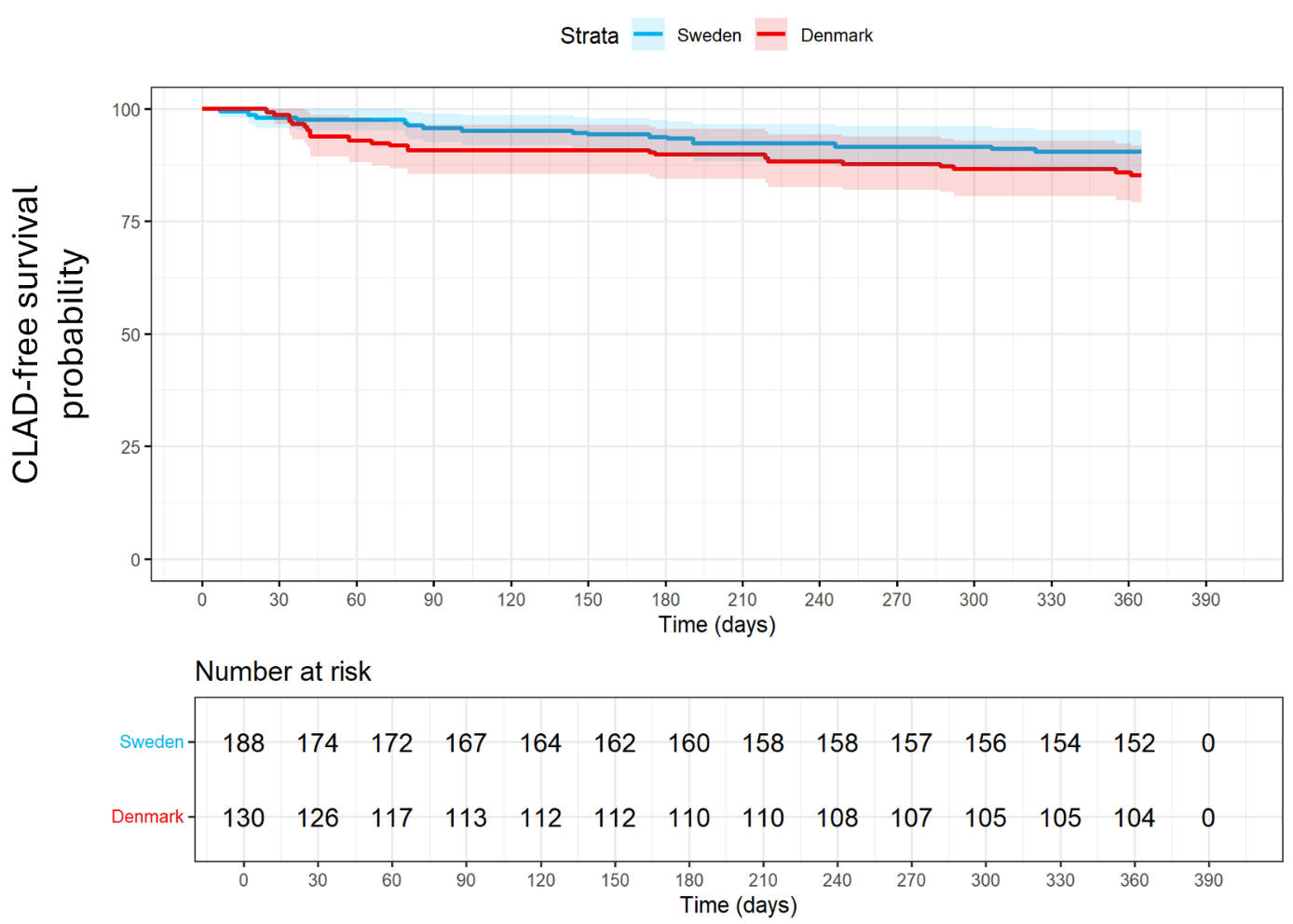


**Supplemental figure 1: No difference in the risk of CLAD development or progression between Sweden and Denmark.** Kaplan Meier curve displaying CLAD-free survival (including survival free of CLAD progression) probability with 95% CI for patients with COVID-19 in Sweden versus in Denmark (blue = Sweden, red = Denmark). There is no significant difference in risk when comparing Swedish and Danish patients with COVID-19 (p = 0.187). A table with numbers at risk for every thirty days is placed below the curve. *CLAD: chronic lung allograft dysfunction, CI: confidence interval, COVID-19: coronavirus disease 2019.*


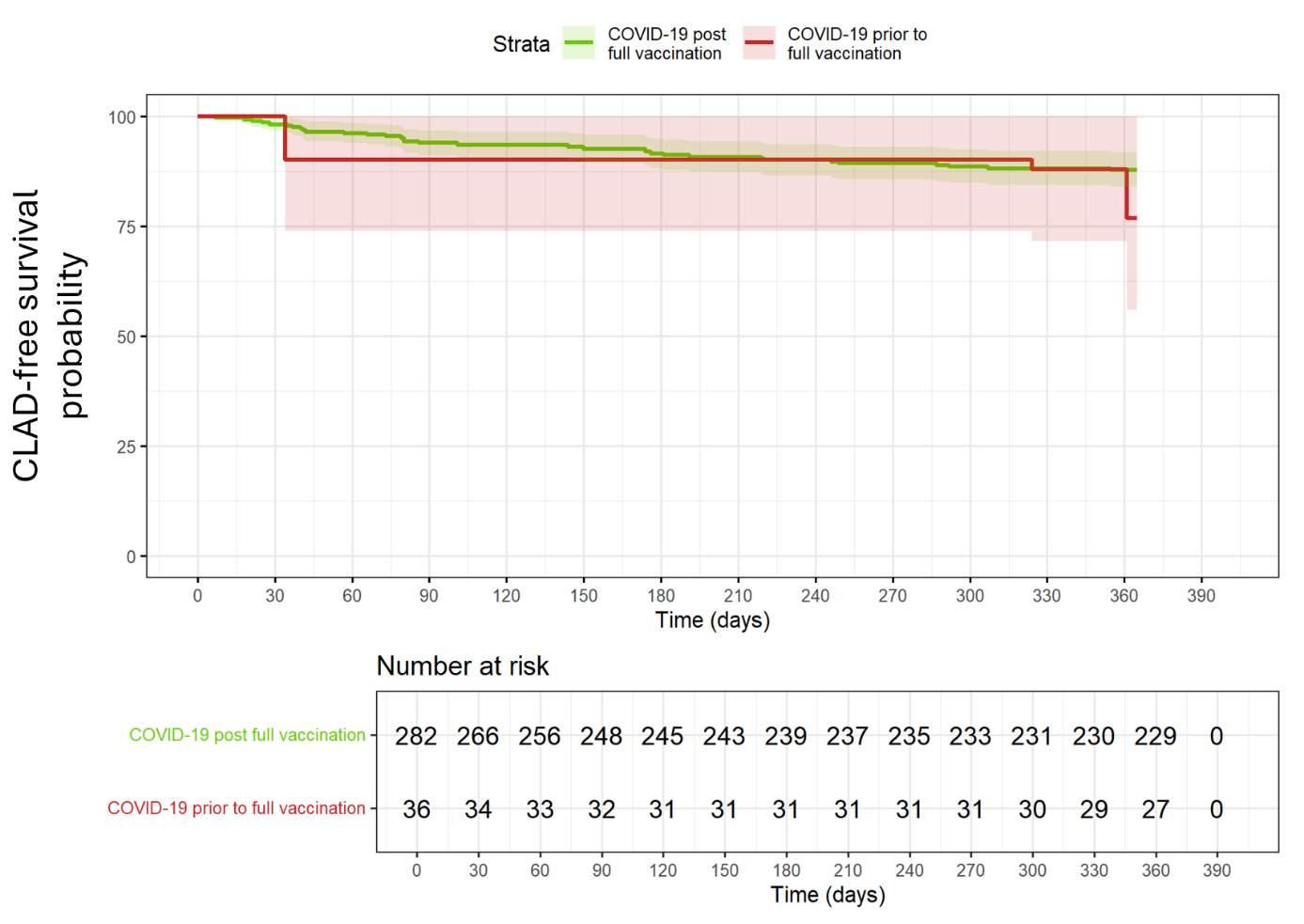


**Supplemental figure 2: No difference in the risk of CLAD development or progression between vaccinated and unvaccinated patients.** Kaplan Meier curve displaying CLAD-free survival (including survival free of CLAD progression) probability with 95% CI for patients with COVID-19 before versus after full vaccination (red = unvaccinated, green = vaccinated). There is no significant difference in risk when comparing unvaccinated patients to vaccinated patients (p = 0.303). A table with numbers at risk for every thirty days is placed below the curve. *COVID-19: coronavirus disease 2019, CLAD: chronic lung allograft dysfunction, CI: confidence interval.*


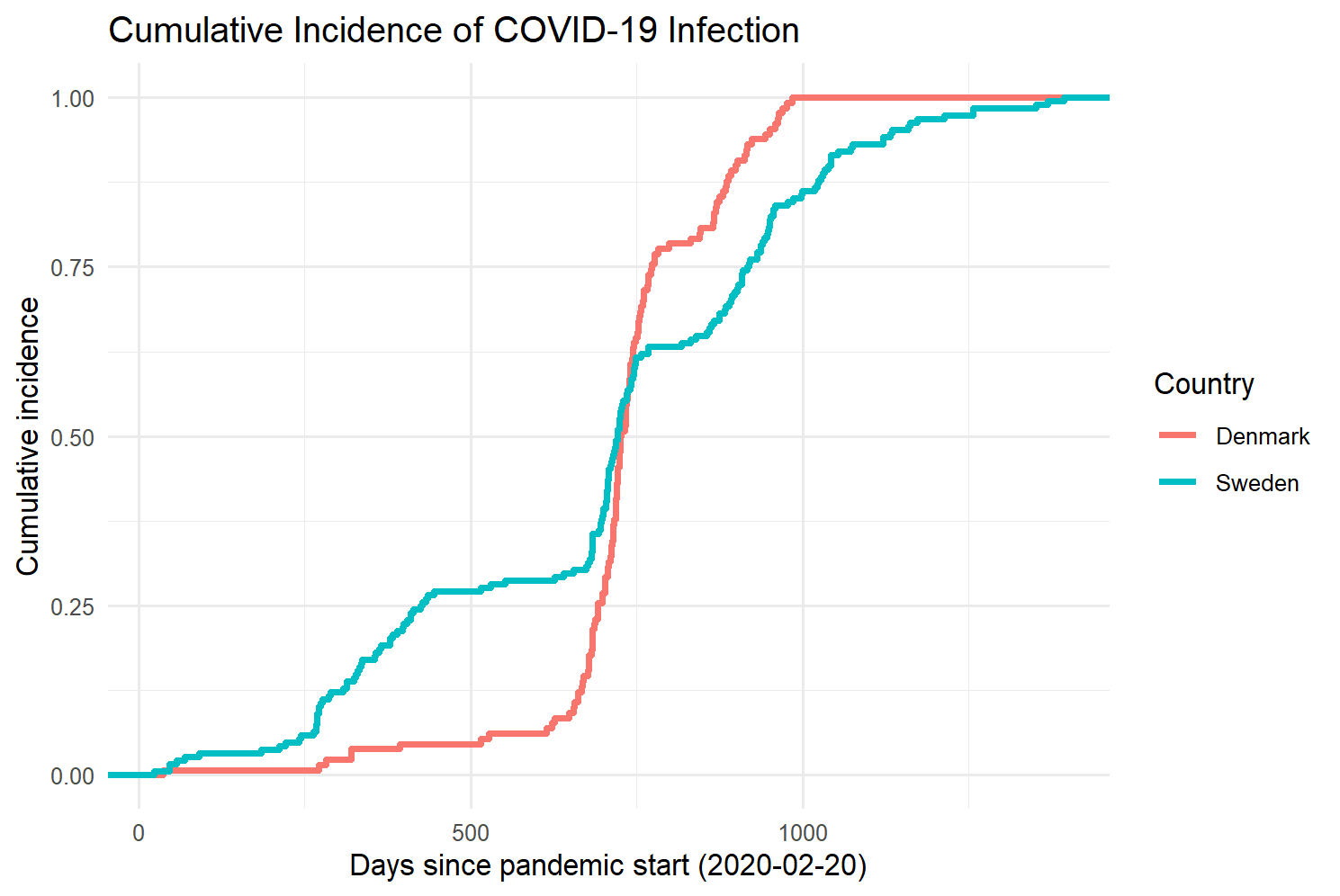


**Supplemental figure 3: Cumulative incidence plot divided by country.** Plot displaying cumulative incidence by days post start of the pandemic (red = Denmark, blue = Sweden). *COVID-19: coronavirus disease 2019.*


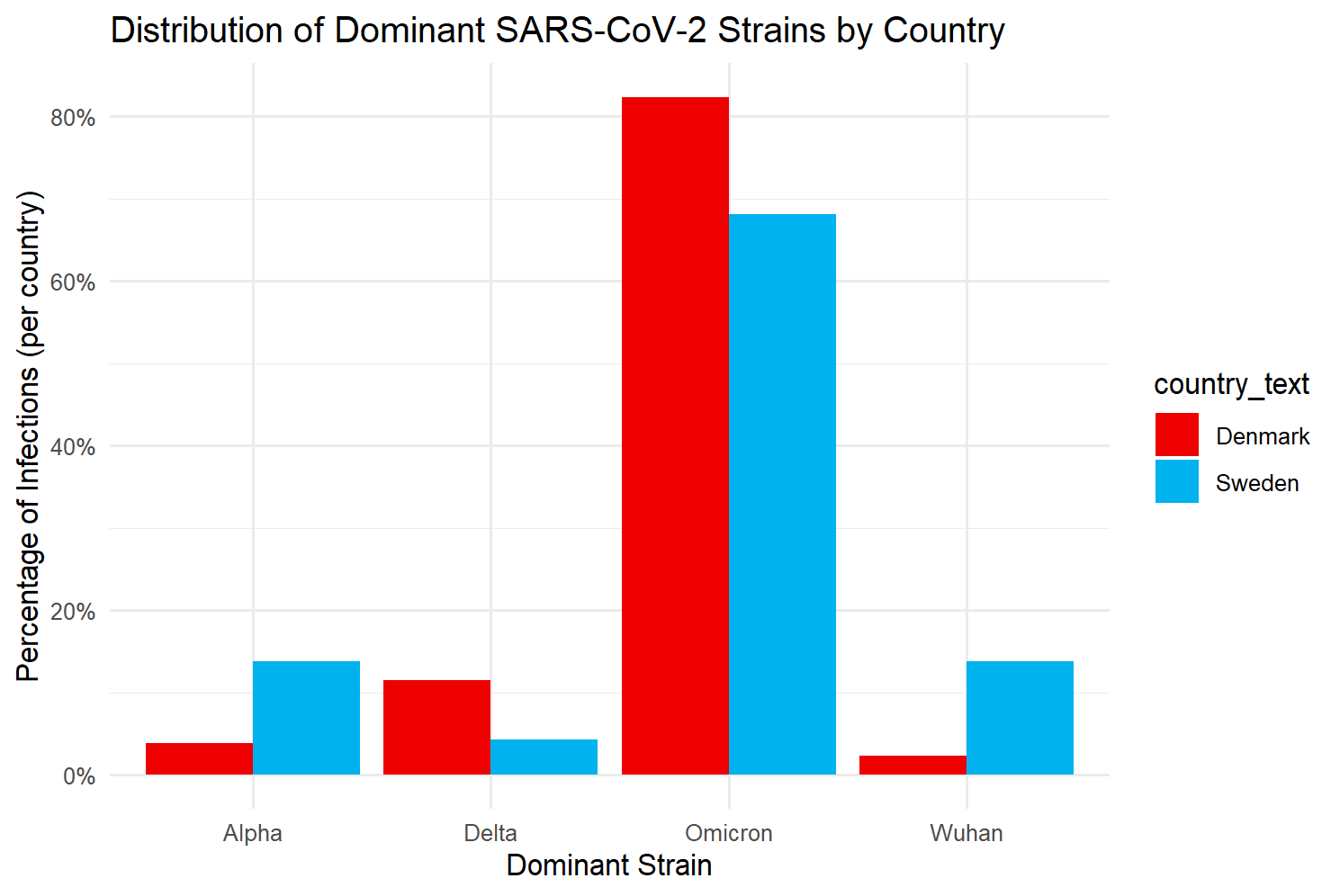


**Supplemental figure 4:** Distribution of dominant SARS-CoV-2 strains among infected lung transplant recipients in Sweden and Denmark. Each bar represents the proportion of infections attributed to a given dominant strain (Wuhan, Alpha, Delta, or Omicron) within each country. Infection date was used as a proxy for viral strain, based on national surveillance timelines. *SARS-CoV-2: severe acute respiratory syndrome coronavirus 2*.


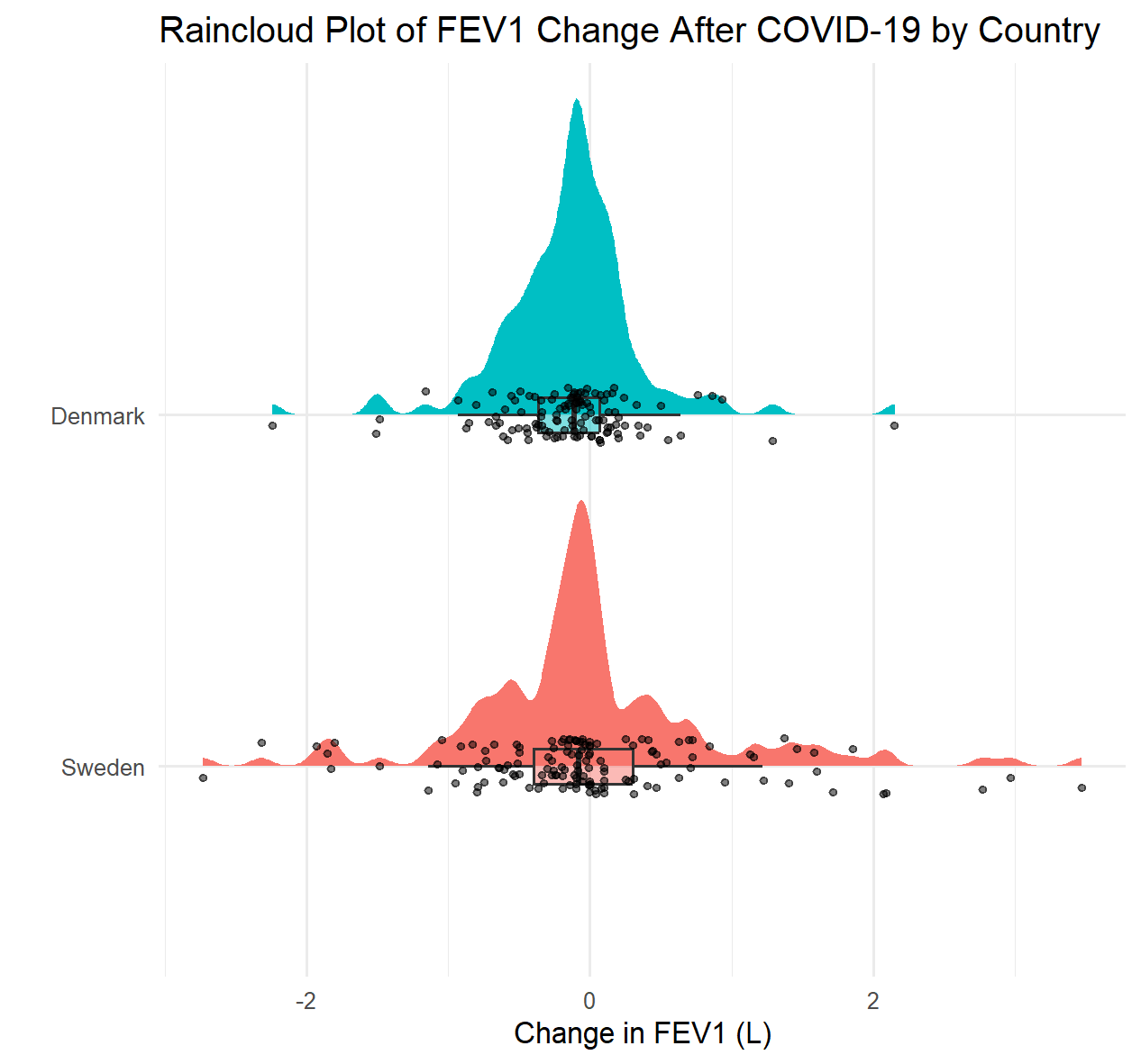


**Supplemental figure 5:** Raincloud plot showing individual-level changes in FEV1 (ΔFEV1) following infection with COVID-19 among LTx recipients in Sweden and Denmark. The plot includes a half-violin (density), boxplot (IQR and median), and raw jittered data for each country. While the mean change in FEV1 did not differ significantly between countries (p = 0.117), Levene’s test indicated a significantly greater variance in ΔFEV1 among Danish patients (p < 0.001), suggesting more heterogeneous post-COVID functional outcomes.
